# Noninvasive In Vivo Estimation of Blood-Glucose Concentration by Monte Carlo Simulation

**DOI:** 10.3390/s21144918

**Published:** 2021-07-19

**Authors:** Chowdhury Azimul Haque, Shifat Hossain, Tae-Ho Kwon, Ki-Doo Kim

**Affiliations:** Department of Electronics Engineering, Kookmin University, Seoul 02707, Korea; c_azimul@kookmin.ac.kr (C.A.H.); shifathosn@kookmin.ac.kr (S.H.); kmjkth@kookmin.ac.kr (T.-H.K.)

**Keywords:** blood-glucose concentration, photoplethysmography (PPG), noninvasive, bio-optical properties, Monte Carlo simulation

## Abstract

Continuous monitoring of blood-glucose concentrations is essential for both diabetic and nondiabetic patients to plan a healthy lifestyle. Noninvasive in vivo blood-glucose measurements help reduce the pain of piercing human fingertips to collect blood. To facilitate noninvasive measurements, this work proposes a Monte Carlo photon simulation-based model to estimate blood-glucose concentration via photoplethysmography (PPG) on the fingertip. A heterogeneous finger model was exposed to light at 660 nm and 940 nm in the reflectance mode of PPG via Monte Carlo photon propagation. The bio-optical properties of the finger model were also deduced to design the photon simulation model for the finger layers. The intensities of the detected photons after simulation with the model were used to estimate the blood-glucose concentrations using a supervised machine-learning model, XGBoost. The XGBoost model was trained with synthetic data obtained from the Monte Carlo simulations and tested with both synthetic and real data (*n* = 35). For testing with synthetic data, the Pearson correlation coefficient (Pearson’s r) of the model was found to be 0.91, and the coefficient of determination (R^2^) was found to be 0.83. On the other hand, for tests with real data, the Pearson’s r of the model was 0.85, and R^2^ was 0.68. Error grid analysis and Bland–Altman analysis were also performed to confirm the accuracy. The results presented herein provide the necessary steps for noninvasive in vivo blood-glucose concentration estimation.

## 1. Introduction

Glucose is a type of sugar that serves as the primary source of energy in the body. Thus, blood glucose refers to the sugar carried by the bloodstream to all the cells in the body for supplying energy. This glucose is derived by consuming food and drink, and the body also releases stored glucose from the liver and muscles. The human body controls blood-glucose concentrations to maintain adequate levels to supply energy uniformly throughout the day. While the amount of blood glucose available should be enough to fuel cells, excessive amounts can strain the circulatory system. Diabetes mellitus is a serious disease that is directly related to the amount of glucose present in the blood. Internationally, blood-glucose levels are specified in terms of the molar concentration and measured in either millimoles per liter or milligrams per deciliter. Excessive amounts of glucose (>140 mg/dL) in the blood causes hyperglycemia, whereas low amounts of glucose (<71 mg/dL) causes hypoglycemia [1].

Continuous blood-glucose measurement is essential as it allows diabetics and normal people to plan a healthy lifestyle. However, invasive and continuous blood-glucose measurements may cause pain and burden to individuals and deter continuous monitoring [2]. Therefore, noninvasive in vivo blood-glucose measurements can overcome the above limitations and prevent pain. However, noninvasive blood-glucose evaluation frameworks for self-monitoring have plenty of room for improvement and are still far from suitable for at-home use.

Photoplethysmography (PPG) is the primary optical method used to distinguish changes in blood volume in the peripheral circulation; it is a cost-effective and noninvasive strategy to estimate biological parameters from the skin surface. PPG is a promising technique for early detection of various atherosclerotic pathologies [3], and the signals require only a few electronic components for recording: a light source that illuminates the skin and a photodetector (PD) that is placed on the same or opposite side to the light source for receiving the light. Depending upon the light source and PD placement, the PPG signals can be divided into two types: reflection and transmission. If the light source and PD are placed on the same side, it is regarded as the reflection mode; if the light source and PD are placed on the opposite side, it is regarded as the transmission mode [4]. The PPG signal consists of AC and DC components. The AC components change with each heartbeat, whereas the DC components, which are also called slow-varying baselines, are composed of low-frequency fluctuations [5]. Over the past few decades, PPG signals have been used for various clinical evaluations, such as monitoring blood oxygenation, blood pressure, and heart rate variability. Studies on measurements of arterial blood oxygenation (SpO_2_) [6] and blood pressure [7] using PPG signals have been reported. Recently, wearable devices have become promising methods of collecting PPG signals to evaluate health information. In [8], the authors successfully developed a wearable printed circuit board (PCB) to collect reflection-type PPG signals and to measure heart rate and SpO_2_. Sen Gupta et al. [9] developed a PPG data acquisition device to record both transmission- and reflection-type signals, and a set of features related to blood glucose levels were extracted from the signals to estimate blood-glucose concentration using their machine-learning algorithm. Furthermore, other health-related parameters such as glycated hemoglobin (HbA1c) can be measured from the PPG signals. In [10], the authors developed gray-box models to estimate HbA1c using digital volume pulse waveforms, which are also called fingertip PPG signals. Monte-Moreno et al. [11] used various physiological parameters, such as heart rate, vascular compliance, blood viscosity, and respiratory frequency, to analyze PPG waveforms such that these signals could later be used for estimating blood glucose; they used support vector machine (SVM), random forest, linear regression, and a neural network classifier for blood-glucose level classifications and reported that the random forest approach produced the best results with an R^2^ value of 0.9.

When a photon is ejected from a light source, it is reflected by the tissue components and returns to the PD or is transmitted through the tissue and reaches the PD placed on the opposite side of the photon source. The PPG signal follows the transmission or reflection mode and detects changes in the blood volume. In our experiments, we examined photon propagation through a finger model via Monte Carlo (MC) simulation by considering blood as a static component and studied the changes in blood volume.

MC simulation is a computational technique that involves random sampling of an actual amount. It is an adaptable technique for simulating photon propagation in biological tissues. The simulation depends on the random walks that photons make as they travel through the tissue, which is chosen by sampling the probability distributions for step size and angular deflection per scattering event. Within the common strategy of MC modeling, light transport in tissues is simulated by following a random walk process that each photon packet undergoes within the tissue model [12]. For each dispatched photon packet, an initial weight is allotted before entering the tissue model. The absorption coefficient $\mu_{a}$ (cm^−1^) and scattering coefficient $\mu_{s}$ (cm^−1^) are used to depict the probability of absorption and scattering, respectively, for a unit path length [13]. The anisotropy factor *g*, which is characterized as the normal cosine of the scattering angle, determines the probability distribution of the scattering angles for first-order approximation. Moreover, the refractive index *n* change between any two regions in the tissue model or at the air–tissue interface determines the angle of refraction. After traveling within a given medium, a fraction of the photon packet exits from the same side of the tissue model; this fraction is calculated as the part of the incident light that is scored as the received light intensity (weight). Interestingly, the negligible portion of the photon packet weight that travels through the medium and exits on the opposite side of the model is scored as the transmittance [14]. In this study, MC simulations were used to infer photon transport within the finger tissue model, and the amount of photons that reach the PD was analyzed to deduce the relationship between the received light intensity and blood-glucose concentration.

In recent years, light transport in a tissue medium has been used for in vivo estimation of health-related parameters, such as blood pressure, blood-glucose concentration, and oxygen saturation in the blood [15]. MC simulations are considered the gold standard for photon migration in the tissue model to noninvasively estimate the health parameters [16]. Noninvasive blood-glucose estimation is a new research trend, and only a few works have reported results with the MC method. Liu et al. [17] introduced a backpropagation MC (BpMC) approach to retrieve the bio-optical properties of multilayered tissues from transmitted and reflected light signals. These properties were used to estimate the blood-glucose concentrations through two types of models: BpMC-DEE and BpMC-CNN. In [18], frequency-modulated continuous wave (FMCW) LIDAR technology was proposed to estimate blood-glucose concentrations, and MC simulations were performed to investigate the feasibility of the method; this approach mainly comprised a near-infrared tunable semiconductor laser and an integrated detector. The glucose concentration was deduced from the slope of the FMCW signal spectrum by analyzing the relationship between the signal intensity and light transit time depending on beat frequency. Enejder et al. [19] reported the use of Raman spectroscopy for quantitative, noninvasive blood-glucose measurements; they tested 17 healthy human subjects and collected 461 sets of Raman spectra transcutaneously along with glucose reference values. Further, a partial least-squares calibration and leave-one-out cross-validation were used for each subject. They reported an R^2^ value of 0.83 ± 0.1. Fluorescence-based glucose sensors were introduced in [20] for in vivo blood-glucose estimation by new receptor systems for glucose recognition and utilization of transduction schemes. According to a mathematical model in this strategy, the acquired optical signals were applied to evaluate glucose concentrations, and the assessments were performed on raw optical signals. However, a simple mathematical model cannot consider the logical relationships between optical signals and physiological parameters; this often creates obstacles for the proposed method to be implemented in clinical scenarios.

In this paper, we propose an MC photon simulation-based model for estimating blood-glucose concentrations through PPG in a finger model. The finger model was used with light at wavelengths of 660 nm and 940 nm, as well as the corresponding bio-optical properties of the finger layers and calculated intensity of the received light. Thereafter, a supervised machine-learning algorithm was used to estimate the blood-glucose concentration using the calculated light intensities and SpO_2_ values. We collected PPG data from 35 volunteers for light at both wavelengths, along with the blood-glucose concentrations and SpO_2_ values as a reference to evaluate the proposed model.

The remainder of this paper is organized as follows. In Section 2, the methodology of this study, including the finger model, deduction of the bio-optical properties, MC photon simulation model, data acquisition procedure, and machine-learning model for estimating blood-glucose concentration are presented. In Section 3, the bio-optical properties of the finger layers, MC photon simulation results, and machine-learning model results are presented. Finally, in Section 4 and Section 5, the discussion and conclusion of the study are presented, respectively.

## 2. Methodology

**Figure 1:**
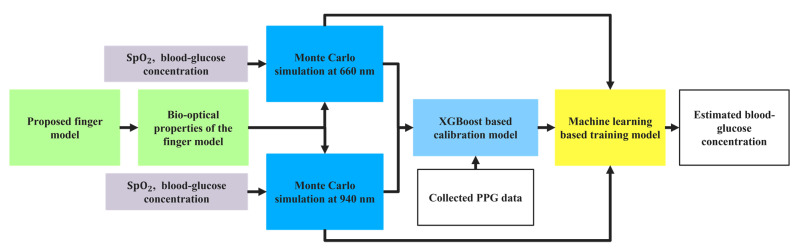
Workflow diagram in this study.

The workflow diagram for the proposed approach is shown in Figure 1 and discussed sequentially.

### 2.1. Proposed Finger Model

**Figure 2:**
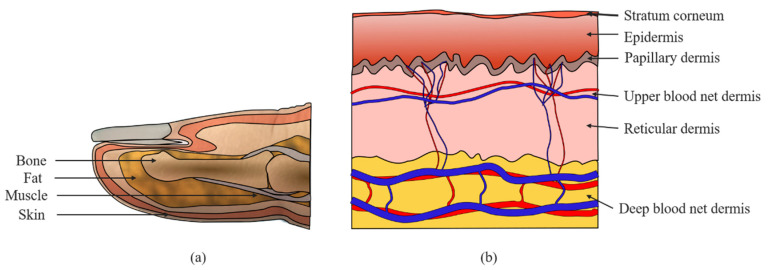
Proposed finger model: (**a**) layers of the finger and (**b**) sublayers of the skin.

For photon transport by MC simulations, a heterogeneous finger model was considered. In the finger model, the spatial distribution of blood and chromophores vary with depth. However, the anatomical constituents of the skin, including skin cell composition, chromophore content, and blood concentration, are generally constant and can be identified. This permits approximation of the skin as a multilayered medium. Before dividing the skin into the subdermal layers, four general layers are considered for the finger model, as shown in Figure 2a. The skin layer, as shown in Figure 2b is divided into six subdermal layers, namely, stratum corneum, epidermis, papillary dermis, upper blood net dermis, reticular dermis, and deep blood net dermis. The first layer of the skin, which is approximately 20 µm thick, is known as the stratum corneum. The second layer is the epidermis and is approximately 80 µm thick. The epidermis contains primarily living cells, a fraction of dehydrated cells, laden cells with keratohyalin granules, columnar cells, melanin dust, small melanin granules, and melanosomes, which are not specifically perfused into the blood [21]. The other four dermal layers with varying volumes of blood are the papillary dermis (150 µm thick), upper blood net dermis (80 µm thick), reticular dermis (1500 µm thick), and deep blood net dermis (100 µm thick). Fat, muscle, and bone are structured beneath the skin layer; the fat layer is 0.55 mm thick, and blood components are absent in this layer. Table 1 summarizes the thicknesses of the layers, including the subdivided skin layers considered in this work.

### 2.2. Bio-Optical Properties of the Finger Model

When light enters a tissue, photons are either absorbed in the media, scattered from the surface, or scattered within the tissue medium. Therefore, the bio-optical properties of the proposed finger model layers are needed for the MC photon simulations and to determine the photon intensities. Accounting for the blood concentration in the tissue is another vital step in measuring the optical properties of the model. The blood is concentrated in a layer measuring 0.05–0.1 mm; thus, the dermis must be divided into four layers in addition to the stratum corneum and epidermis [22]. Generally, the absorption coefficient $\mu_{a}$, scattering coefficient $\mu_{s}$, anisotropy *g*, and refractive index *n* are considered as the optical properties of tissues.

The general equation of the absorption coefficient $\mu_{a}$ for a heterogeneous biological tissue can be represented as Equation (1).

(1) $$\mu_{a}\left(\lambda \right)=\sum_{i=1}^{k}\left(\mu_{a}{}_{i}\left(\lambda \right)\times V_{i}\right)+\mu_{a}^{\left(0\right)}\left(\lambda \right)\times \left(1-\sum_{i=1}^{k}V_{i}\right)$$

where $V_{i}$ is the volume fraction of the *i*-th skin layer, and *k* is the total number of layers; $\mu_{a}^{\left(0\right)}\left(\lambda \right)$ is the baseline absorption coefficient and can be expressed as Equation (2).

(2) $$\mu_{a}^{\left(0\right)}\left(\lambda \right)=7.84\times 10^{7}\times \lambda^{-3.255}$$

Given $\mu_{a}^{\left(0\right)}\left(\lambda \right)$, the absorption coefficient of the *i*-th dermal sublayers at a wavelength λ can be written as

(3) $$\mu_{a_{i}}\left(\lambda \right)=\left(V_{Art_{i}}\times \mu_{a_{Art_{i}}}\left(\lambda \right)\right)+\left(V_{Ven_{i}}\times \mu_{a_{Ven_{i}}}\left(\lambda \right)\right)+\left(V_{wat_{i}}\times \mu_{a_{wat_{i}}}\left(\lambda \right)\right)\;+\left[1-\left(V_{Art_{i}}+V_{Ven_{i}}+V_{wat_{i}}\right)\right]\times \mu_{a}^{\left(0\right)}\left(\lambda \right)$$

where *V_Art_* and *V_Ven_* are the arterial and venous blood-volume fractions, respectively; $\mu_{a_{Art}}$, $\mu_{a_{Ven}},$ and $\mu_{a_{wat}}$ represent the absorption coefficients of arterial blood, venous blood, and water, respectively.

Only melanin and water were considered to calculate the absorption coefficients of the stratum corneum and epidermis because these layers do not contain any blood cells. The absorption coefficient of the epidermis can be expressed as follows [23]:

(4) $$\mu_{a}^{\left(Epi\right)}\left(\lambda \right)=(V_{m}\times \mu_{a}{}_{m}\left(\lambda \right))+\left(V_{w}\times \mu_{a_{wat}}\left(\lambda \right)\right)+\left[1-\left(V_{m}+V_{w}\right)\right]\times \mu_{a}^{\left(0\right)}\left(\lambda \right)$$

(5) $$\mu_{a}{}_{m}\left(\lambda \right)=6.6\times 10^{10}\times \lambda^{-3.3}$$

where $V_{m}$ is the melanin volume fraction, which is considered as 10%, $\mu_{a}{}_{m}\left(\lambda \right)$ is the absorption coefficient of melanin at wavelength λ, $V_{w}$ is the water volume fraction in the epidermis, and $\mu_{a_{wat}}\left(\lambda \right)$ is the absorption coefficient of water at wavelength λ.

The equation for calculating the absorption coefficient of the stratum corneum is adopted from [24] and expressed as

(6) $$\mu_{a}^{\left(StC\right)}\left(\lambda \right)=\left[\left(0.1-0.3\times 10^{-4}\lambda \right)+0.125\times \mu_{a}^{\left(0\right)}\left(\lambda \right)\right]\times \left(1-V_{w}\right)+V_{w}\mu_{a_{Wat}}\left(\lambda \right)$$

The absorption coefficients of arterial and venous blood can be calculated from oxygen saturation [25,26]. In the finger model, the four subdivided dermal layers contain blood, and hence, glucose. Therefore, glucose is present in the arterial and venous blood of these four dermal layers, whose absorption coefficients can be expressed as follows:

(7) $$\mu_{a_{Art}}\left(\lambda \right)=\left(SaO_{2}\times \mu_{a_{HbO}}\left(\lambda \right)\right)+\left(\left(1-SaO_{2}\right)\times \mu_{a_{HHb}}\left(\lambda \right)\right)+(\epsilon_{g}\left(\lambda \right)\times c_{g}\left(\lambda \right))$$

(8) $$\mu_{a_{Ven\;}}\left(\lambda \right)=(SvO_{2}\times \mu_{a_{HbO}}\left(\lambda \right))+\left(\left(1-SvO_{2}\right)\times \mu_{a_{HHb}}\left(\lambda \right)\right)+(\epsilon_{g}\left(\lambda \right)\times c_{g}\left(\lambda \right))$$

where $\mu_{a_{HbO}}$ and $\mu_{a_{HHb}}$ represent the absorption coefficients of oxyhemoglobin and deoxyhemoglobin, respectively; $SaO_{2}$ and $SvO_{2}$ are the arterial and venous oxygen saturation values, respectively; $\epsilon_{g}$ is the molar absorption coefficient (L·mol^−1^·cm^−1^), and $c_{g}$ is the molar concentration (mol·L^−1^) of glucose in the arterial and venous blood of the dermal layers. To calculate the absorption coefficients of the venous and arterial blood, $SvO_{2}$ is considered to be 10% lower than $SaO_{2}$ [27]. The baseline blood volume fraction ($V_{b}$) and water volume fraction ($V_{wat}$) of the proposed finger layers for calculating the bio-optical properties are obtained from [28,29,30] and listed in Table 2.

The absorption coefficients of water, oxyhemoglobin, and deoxyhemoglobin are adopted from other studies [31,32,33] and listed in Table 3. As we used two wavelengths of light (660 nm and 940 nm) in the simulation model, the absorption coefficients of water, oxyhemoglobin, and deoxyhemoglobin are listed for these two wavelengths. The molar absorption coefficient ($\epsilon_{g}$) of glucose for 660 nm and 940 nm are 0.0002 and 0.001 L·mol^−1^·cm^−1^, respectively [34].

### 2.3. Monte Carlo Simulation Model for Photon Propagation

**Figure 3:**
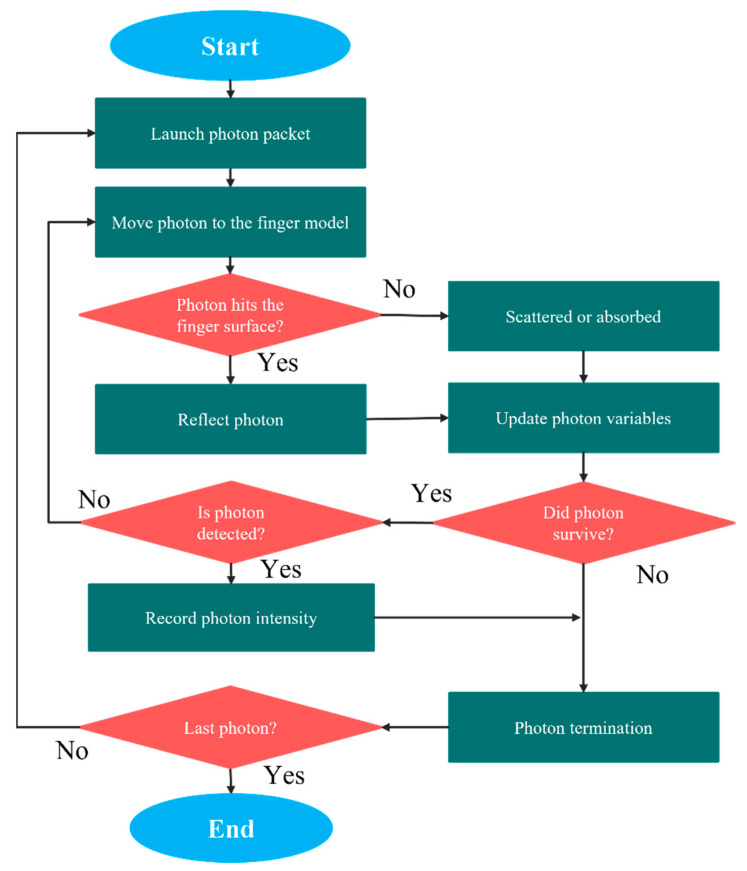
Flowchart for MC simulation in the finger model.

Photon propagation with MC simulations is an adaptable yet thorough method of handling photon transport simulations. In our experiment, the MC simulation was used to deduce light propagation in the finger model. The voxel-based MC algorithm [35] was considered for accurate and efficient photon transport modeling. The 3D multilayered volume, where a single integer was assigned to each voxel to indicate the index of the layer, was designed for two light wavelengths (660 nm and 940 nm). The bio-optical properties of the layers considered for the finger model for 660 nm and 940 nm were assigned to each layer of the volume separately for photon propagation in the model. Once a photon package enters the heterogeneous finger model, it is randomly scattered or absorbed by the layers. The remaining photons that reach the detector are collected, and their intensity is analyzed to estimate the blood-glucose concentration. The flowchart of the MC simulation for light propagation in the heterogeneous finger model is shown in Figure 3.

Initially, a photon packet was launched into the proposed finger model, and the initial weight was considered as 1 (one). The step size (*l*) was calculated by random sampling of the probability of photon scattering [36]. In this experiment, after the photon entered the finger model, we evaluated whether the photon contacted the surface of the finger. Once the photon hits the surface of the finger, it is reflected, and its variables are updated. The position vector, direction vector, and weight of the photon were considered as the photon variables and were updated after reflection. The intensities of the remaining photons were recorded after detection by the photon detector. Absorption and scattering were expected to have occurred in the case of free photon propagation and that the photon packet was oriented through randomly generated deflections and azimuth angles. The Henyey–Greenstein phase function [37] was used to calculate the scattering angles θ while generating the azimuth angles randomly in the range of 0 to 2 π. The cosine of the scattering angle can be expressed as

(9) $$cos\theta =\{\begin{array}{c} \frac{1}{2g}\left[1+g^{2}-{\left(\frac{1-g^{2}}{1-g+2g\xi }\right)}^{2}\right]\;if\;g\neq 0\\ 1-2\xi \;\;\;\;\;\;\;\;if\;g=0 \end{array}$$

where g is known as the scattering anisotropy; g=0 indicates isotropic scattering, whereas g=1  indicates scattering that is primarily in the forward direction. The step size (*l*) depends on a random number ξ (0 < ξ < 1), absorption coefficient ($\mu_{a})$, and scattering coefficient ($\mu_{s}$) and can be expressed as Equation (10).

(10) $$l=-\frac{ln\xi }{\mu_{a}+\mu_{s}}$$

Our model was examined in reflection mode. The optical source and detector were placed 0.4 mm apart, and the intensities of the detected photons were recorded, which is referred to as the mean weight of the detected photon packets. The relationship among penetration depth, optical path, and source-detector separation in the reflectance geometry were scrutinized using this model. The mean of the total simulated path length of the photon packets from the source to the detector was used to calculate the mean optical path. This simulation was repeated until the desired number of photon packets were detected to estimate the blood-glucose concentration from the photon intensity.

### 2.4. Machine-Learning Model and Blood-Glucose Concentration Estimation

**Figure 4:**
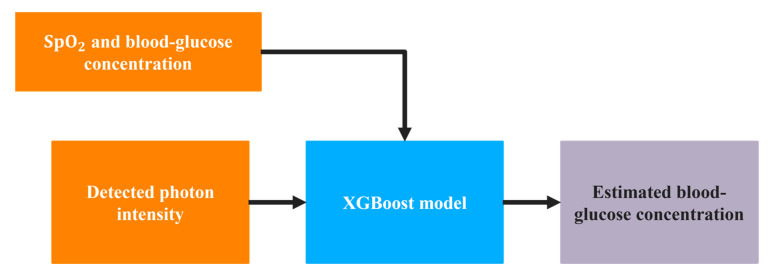
Workflow diagram for estimating blood-glucose concentration.

As shown in Figure 4, after calculating the photon intensity from MC simulations at 660 nm and 940 nm, the values were used as one of the inputs to a supervised machine-learning regression model. A powerful approach for the supervised regression model, XGBoost, was used in this study. Along with the light intensity, the SpO_2_ value was also used as the input to the regression model, with blood-glucose concentration as the target of the model. The ranges of SpO_2_ and glucose values used in the regression model are listed in Table 4.

After simulation, approximately 1581 data samples were obtained for the stated range of glucose concentrations and SpO_2_, and these samples were considered as the synthetic data for training the machine-learning model.

XGBoost parameters used for the regression include learning rate = 0.3, maximum depth = 6, and number of estimators = 100. Five evaluation matrices were used to check the accuracy of the glucose concentration estimations, namely, mean-squared error (MSE), mean absolute error (MAE), root mean-squared error (RMSE), coefficient of determination (R^2^), and Pearson correlation coefficient (Pearson’s r). Clark’s error grid analysis (EGA) [38] and the Bland–Altman plot were used to visualize the estimated values and corresponding estimation errors, respectively.

### 2.5. Data Acquisition

**Figure 5:**
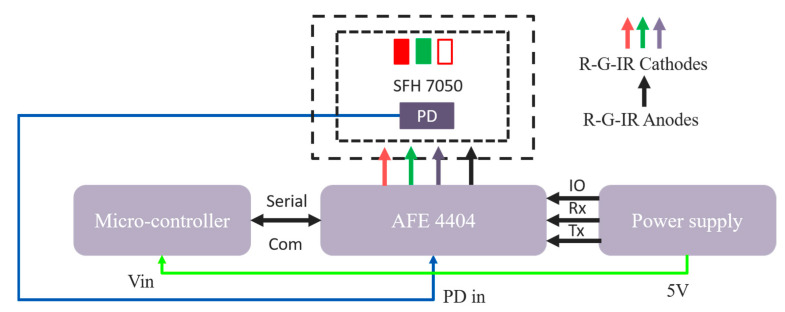
Block diagram of proposed hardware for data acquisition.

For collecting PPG signals from the subjects, a hardware system was proposed. The ESP32-PICO-V3 [39] was used as a processing unit for controlling the entire system. This microcontroller has an on-chip RF communication system so that the data can be transmitted to a remote server without any external communication module. A surface-mounted device (SMD) module, SFH 7050 [40], was used for collecting the reflection mode PPG signal. This module contains three different light emitting diodes: green, red, and infrared and a photodetector (PD). This photodetector can respond in the spectral range of sensitivity of 400 nm to 1100 nm. A bio-sensing analog front end (AFE), AFE 4404 [41], was used to control the LEDs and the PD. The three transmitter pins (Tx1, Tx2, Tx3) of AFE 4404 were designated for the three LEDs. In our study, we considered the PPG signals that were collected with the red and infrared LED. Figure 5 illustrates the block diagram of our proposed hardware for data acquisition.

**Figure 6:**
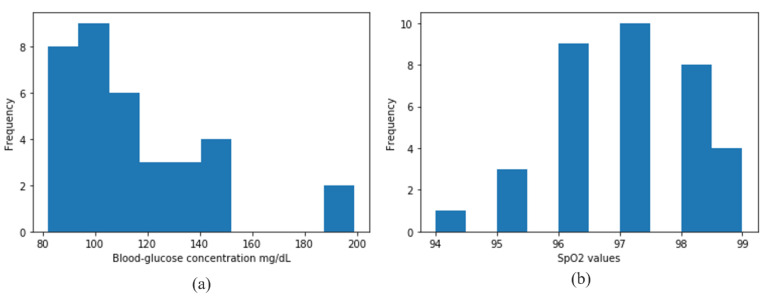
Histograms of (**a**) blood-glucose concentration and (**b**) SpO_2_ values of the subjects.

Thirty-five subjects voluntarily provided data for this study. From each subject, we collected 240 s of PPG data in the reflection mode. The MC simulation model was designed for 660 nm and 940 nm light, considering the PPG data of red and infrared wavelengths. Along with the PPG signals of the subjects, we also measured their blood-glucose concentration using the Caresens II Plus [42] and their SpO_2_ using another clinical device [43]. Figure 6 illustrates the histograms of the measured blood-glucose concentrations and SpO_2_ values of the subjects.

**Figure 7:**
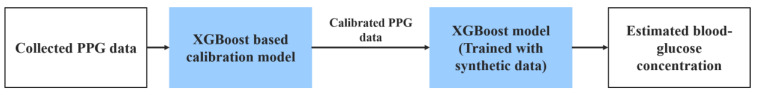
Data calibration block diagram.

After collecting the PPG data from the subjects, we calibrated these data with the synthetic data from the MC simulations. Our collected PPG data had a 22-bit resolution and for the red and infrared wavelengths, the data were stored in the range of 399,000 and 425,000, respectively, using the AFE 4404. We used another XGBoost regressor model for calibrations, where the calibrated PPG data were used in the XGBoost model trained with the synthetic data to estimate the blood-glucose concentrations. Figure 7 depicts the calibration stage and testing steps for estimating blood-glucose concentration.

## 3. Results

### 3.1. Bio-Optical Properties

**Figure 8:**
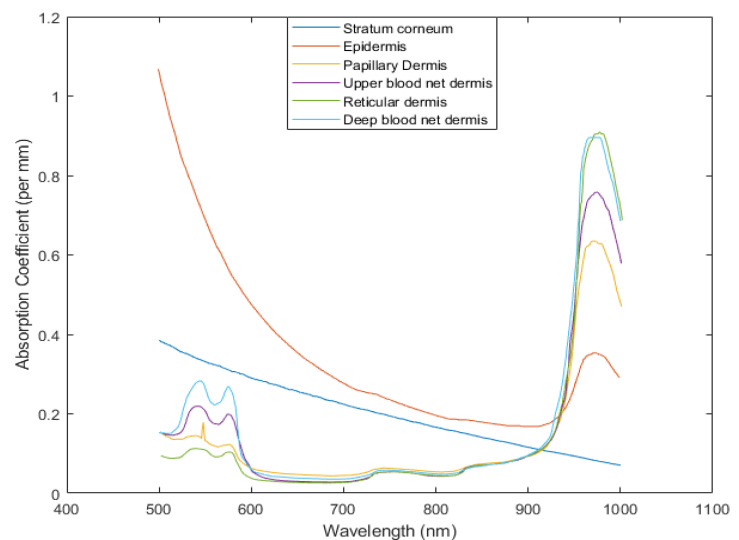
Absorption coefficients of the skin layers as functions of wavelength [24].

The bio-optical properties, i.e., the absorption coefficient, scattering coefficient, anisotropy, and refractive index of the layers of the proposed finger model are necessary to design the MC simulation model for the experiments. The absorption coefficients of the skin layers were calculated using Equations (1)–(8) and Table 1, Table 2 and Table 3 by adopting information from [13,22,27,41]. Figure 8 shows the absorption coefficients of the skin layers in the wavelength range of 500 nm to 1000 nm.

Other optical properties of the finger model layer, i.e., scattering coefficient $\mu_{s}$, anisotropy *g*, and refractive index *n* were adopted from the literature [29,44]. The absorption coefficients of fat, muscle, and bone were also adopted from this literature. As we simulated photons in two wavelengths (660 nm and 940 nm) by MC simulation to estimate blood-glucose concentrations, Table 5 represents the optical properties of the proposed finger model layers for these two wavelengths.

### 3.2. Monte Carlo Simulation Results

**Figure 9:**
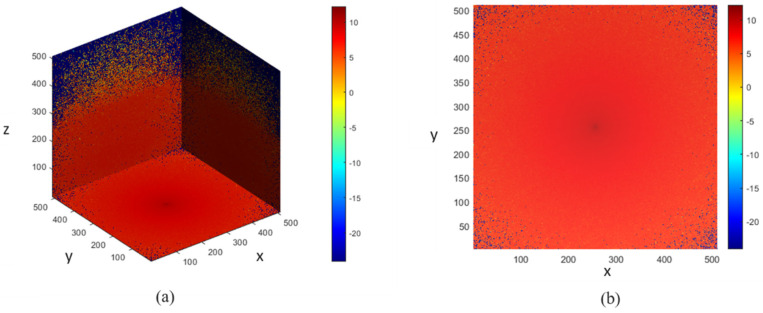
Photon fluence in the voxel-based finger model at 660 nm: (**a**) 3D and (**b**) XY-plane views.

**Figure 10:**
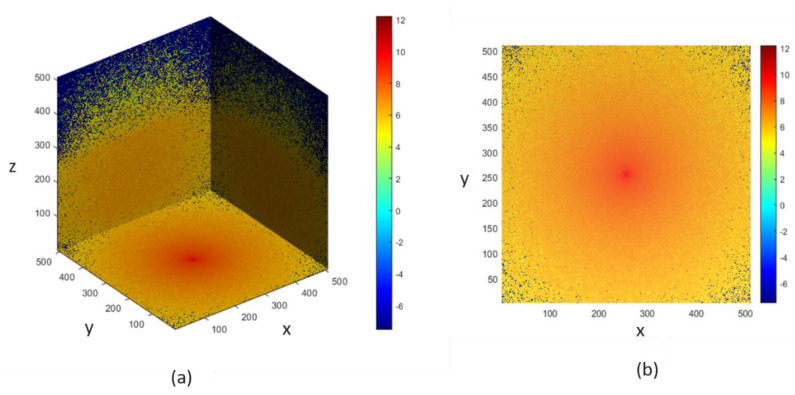
Photon fluence in the voxel-based finger model at 940 nm: (**a**) 3D and (**b**) XY-plane views.

The voxel-based MC simulation model, described in Section 2.3, was designed for photon propagation in the proposed finger model, and the scattering events occurring within the finger model for the two wavelengths were recorded. In Figure 9 and Figure 10, the photon fluence within the heterogeneous finger model is shown for the bio-optical properties at 660 nm and 940 nm. The MC photon migration was performed in a 5.02-mm-thick slab layered in nine parts, and the photon source and detector were placed 4 mm apart. A 64-bit operating system (Windows OS) with 20 GB RAM and an Intel Core i7 CPU was used for the simulations. The designed finger model was contained in 6 × 6 × 6 ${\mathrm{mm}}^{3}$ voxels and required about 5 h to complete the MC simulation. The color bars in the figures illustrate the intensities of the fluence rates throughout the voxel-based finger model.

### 3.3. Estimation of Blood-Glucose Concentration

#### 3.3.1. Model Evaluation with Synthetic Data

**Figure 11:**
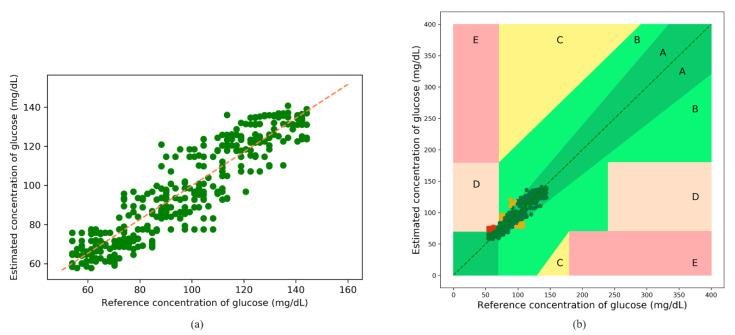
(**a**) Fitted plot after XGBoost regression, and (**b**) error grid analysis (EGA) plot for the synthetic data.

The following results were obtained after blood-glucose concentration estimations using the synthetic data obtained from the MC simulations; the fitted plot after XGBoost regression is shown in Figure 11a. The orange line in the figure indicates a perfect fit. Most of the predicted values are observed to be scattered around the perfect line. Figure 11b illustrates the error grid analysis (EGA), with Zone A (clinically accurate data) containing most of the samples, and Zone B (data outside 20% of the reference but would not lead to inappropriate treatment), and Zone D (indicating potential failure in detecting hyperglycemia or hypoglycemia) containing a negligible number of samples. There are no samples in Zones C and E. Table 6 lists the zonal accuracies of the EGA plots. The unit of estimated blood-glucose concentrations is mol/L but is converted to mg/dL for the EGA plot using Equation (11).

(11) $$1\;\mathrm{mg}\cdot {\mathrm{dL}}^{-1}=0.0555\;\mathrm{mmol}\cdot L^{-1}$$

**Figure 12:**
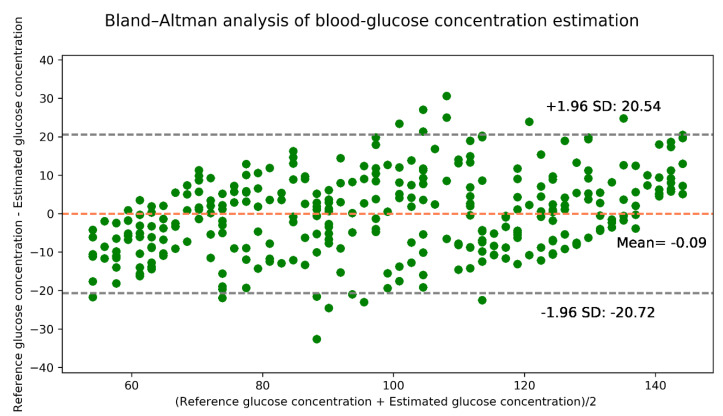
Bland–Altman analysis for the estimated glucose concentrations for the synthetic data.

The Bland–Altman analysis shown in Figure 12 indicates that the estimation model provides a bias of −0.09 ± 10.53 and that the limits of agreement (95%, 1.96 SD) range from −20.72 to 20.54. The values of the evaluation metrics are listed in Table 7.

#### 3.3.2. Model Evaluation with Real Data

**Figure 13:**
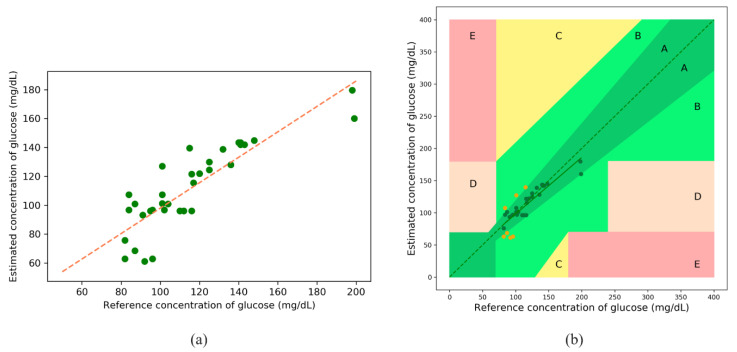
(**a**) Fitted plot after XGBoost regression, and (**b**) EGA plot for real data.

The following results were obtained after blood-glucose concentration estimations using the calibrated PPG data, and the fitted line after XGBoost regression is shown in Figure 13a. The orange line indicates a perfect fit. Figure 13b illustrates the EGA, with Zone A (clinically accurate data) containing most of the samples, and Zone B (data outside of 20% of the reference but would not lead to inappropriate treatment) containing the remaining samples. There are no samples in Zones C, D, and E. Table 8 lists the zonal accuracies of the EGA plot.

**Figure 14:**
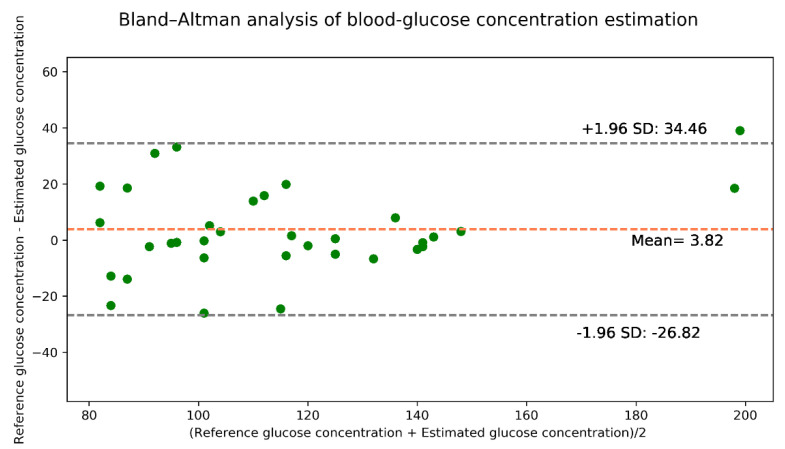
Bland–Altman analysis for the estimated glucose concentration for real data.

The Bland–Altman analysis shown in Figure 14 indicates that the estimation model provides a bias of 3.82 ± 15.63 and that the limits of agreement (95%, 1.96 SD) range from −26.82 to 34.46. The values of the evaluation metrics are listed in Table 9.

We also used our calibrated PPG data for the models presented in [9] for comparisons with the proposed MC simulation model. Table 10 presents the evaluation metrics of this comparison.

In [9], the authors used 17 discriminant features from the collected PPG signals. After segmenting the individual PPG signals for about 3 s, they segmented the signals for red and infrared wavelengths for use with the feature extraction modules of their models. The features are a mixture of PPG-based physiological features, signal-oriented characteristics, and physical parameters, such as zero-crossing rate (ZCR), autocorrelation (ACR), Kaiser–Teager energy (KTE), power spectral density (PSD), autoregressive coefficients (ARC), blood oxygen saturation (SpO_2_), and body mass index (BMI). Further, they considered input features from the spectral analysis of the PPG signals, such as kurtosis (kurt) and skewness (skew) of the frequency distribution. Alongside these features, they calculated the heart rate (HR) and breath rate (BR) to validate the PPG signals. For each frame *f* of the PPG signal *s,* the feature vector is expressed as Equation (12) [9].

(12) $$X_{F}^{f}=\left[\begin{array}{c} s_{zcr},\;s_{ACR},\;s_{PSD}^{kurt},\;s_{PSD}^{var},\;s_{PSD}^{mean},\;s_{KTE}^{kurt},\;s_{KTE}^{var},\;s_{KTE}^{mean},\;s_{KTE}^{skew},\;s_{spec}^{kurt},\;s_{spec}^{skew},\;\\ s_{wavelet}^{mean},\;s_{AR},\;s_{spo2},\;s_{skew},\;s_{sad},\;BMI \end{array}\right]$$

In the case of our model, for each PPG signal *s,* the feature vector can be expressed as Equation (13)

(13) $$X_{F}=\left[S_{SpO2}\right]$$

In Table 10, the Pearson’s r values represent the performances of the models, the number of features denotes the features used in the model, and the number of estimators represents the number of estimator trees in the models. The run time column in Table 10 represents the average execution time for estimating the blood-glucose concentration for one set of inputs.

From Table 10, it can be observed that our model outperforms the random forest model of [9] for all metrics. The Pearson’s r of the XGBoost model [9] is slightly better than ours. However, the XGBoost model of [9] uses more features than ours to estimate blood-glucose concentrations; this renders the model [9] more complex and it requires more time to estimate the blood-glucose concentrations with comparable accuracy. We used only SpO_2_ as the feature to estimate blood-glucose concentration, which reduces the model complexity and renders the model independent of different features.

## 4. Discussion

A Monte Carlo (MC) photon simulation-based model for estimating blood-glucose concentration via PPG on the fingertip is presented in this paper. The MC method was chosen for the photon simulations in the finger model because of its flexibility in computing optical interactions with biological tissues. A heterogeneous finger model with skin, fat, muscle, and bone layers was designed to propagate photons. To facilitate photon simulations in the model, the skin layer was divided into six sublayers. Two of the sublayers do not contain any blood and the remaining sublayers have blood flow. Bio-optical properties such as absorption coefficient, scattering coefficient, anisotropy, and refractive index of these sublayers were obtained at both 660 nm and 940 nm to design the finger model for photon simulations.

After Monte Carlo photon propagation in the finger model, the detected photon intensities were recorded for use in the machine-learning model. The XGBoost regressor was used to analyze the relationships among the parameters. For the inputs to the machine-learning model, we used the detected photon intensities, which were considered as synthetic data, along with specific SpO_2_ and glucose values. Four evaluation metrics, as well as the EGA plot and Bland–Altman analysis were considered to evaluate the MC-based model performance. We also collected PPG data from 35 volunteers with red and infrared light to evaluate the proposed model. By analyzing the blood-glucose estimation results, the proposed model was shown to have better performance than a few comparison models from the literature. The Pearson correlation coefficient (Pearson’s r) and R^2^ values of the synthetic data were 0.91 and 0.83, respectively. For the collected PPG data, the Pearson’s r and R^2^ values were 0.85 and 0.68, respectively. Moreover, the EGA showed clinically accurate results.

Previous studies in the field of noninvasive estimation of blood-glucose concentrations [17,18,19,20] has focused on deriving mathematical relationships between optical signals and physiological parameters, which often creates obstacles in estimating the desired parameters accurately. Moreover, PPG-based physiological features, signal-oriented characteristics, and physical parameters such as zero-crossing rate, autocorrelation, body mass index (BMI) often fail to estimate the blood-glucose concentration more accurately. Therefore, to reduce the possibility of inaccurate estimation of blood glucose concentration, the proposed finger model has been considered as heterogeneous, which was not considered in previous studies, and the bio-optical properties have also been deduced. The heterogenous finger model proposed in our study provides the scope for optimizing the model errors, i.e., the more precise design of the model and more accurate results. As the earlier studies in this field focused on deriving a mathematical model for estimating the blood glucose concentration, there is little scope for optimizing the model error as well as model complexity. Furthermore, in the case of a mathematical model, the computational time for estimating blood glucose concentration is longer than that of our proposed model. The Monte Carlo (MC) photon simulation-based model provides an estimation model that requires less time to estimate the desired parameters, thus reducing the computational time of the system.

In our study, heterogeneous finger models are considered for each layer thickness, but the thickness can vary from person to person. Therefore, future study may include variations in layer thickness and collecting more diverse datasets for both hypoglycemic and hyperglycemic subjects. Despite some limitations of this study, the proposed method is suitable for research purposes.

## 5. Conclusions

In this work, we proposed a Monte Carlo (MC) photon simulation-based model for noninvasive in vivo estimations of blood-glucose concentrations. The proposed approach comprises a finger model for propagating photons with the bio-optical properties of each of the layers at two wavelengths. After photon propagation by MC simulation, we used the detected photon intensities to estimate the blood-glucose concentrations with a supervised machine-learning model. The results were visualized using error grid analysis (EGA) plots and Bland–Altman analyses. Compared with other commonly used noninvasive blood-glucose estimation models, our proposed model was shown to be less complex but more accurate for estimating blood-glucose concentration.

## Figures and Tables

**Table 1:** Thicknesses of the layers of the proposed finger model.

Layer	Thickness (mm)
Stratum corneum	0.02
Epidermis	0.25
Papillary dermis	0.1
Upper blood net dermis	0.08
Reticular dermis	0.2
Deep blood net dermis	0.3
Fat	0.55
Muscle	1.5
Bone	2

**Table 2:** Blood and water volume fractions of the proposed finger model layers.

Layer	$\mathrm{Blood}\;\mathrm{Volume}\;\mathrm{Fraction},\;V_{b}\;(\%)$	$\mathrm{Water}\;\mathrm{Volume}\;\mathrm{Fraction},\;V_{wat}\;(\%)$
Stratum corneum	0	5
Epidermis	0	20
Papillary dermis	5	50
Upper blood net dermis	20	60
Reticular dermis	4	70
Deep blood net dermis	10	70
Fat	0	70
Muscle	0	70
Bone	0	0

**Table 3:** Absorption coefficients of water, oxyhemoglobin, and deoxyhemoglobin.

Element	Absorption Coefficient (mm^−1^)	
	660 nm	940 nm
$\mathrm{Water}\;(\mu_{a_{Wat}}$)	0.00041	0.00181811
$\mathrm{Oxyhemoglobin}\;(\mu_{a_{HbO}}$)	0.0171	0.1728
$\mathrm{Deoxyhemoglobin}\;(\mu_{a_{HHb}}$)	0.065	0.037

**Table 4:** Ranges of SpO_2_ and glucose molar concentration values in the regression model.

	Lower Limit	Upper Limit	Increment
SpO_2_ (%)	70	100	1
Molar concentration (mol·L^−1^)	3.0	8.0	0.1

**Table 5:** Optical properties of the finger model.

Layers	$\mathrm{Absorption}\;\mathrm{Coefficient},\;\mu_{a}$ mm^−1^		$\mathrm{Scattering}\;\mathrm{Coefficient},\;\mu_{s}$ mm^−1^		Anisotropy, *g*	Refractive Index, *n*
	660 nm	940 nm	660 nm	940 nm		
Stratum corneum	0.24959	0.09745	100		0.86	1.5
Epidermis	0.33538	0.21397	45		0.8	1.34
Papillary dermis	0.04624	0.23308	30		0.9	1.4
Upper blood net dermis	0.02898	0.24202	35		0.95	1.39
Reticular dermis	0.02636	0.29906	25		0.8	1.4
Deep blood net dermis	0.03749	0.32549	30		0.95	1.38
Fat	0.0104	0.0170	6.20	5.42	0.8	1.37
Muscle	0.0816	0.0401	8.61	5.81	0.5	1.37
Bone	0.0351	0.0457	34.45	24.70	0.92	1.37

**Table 6:** Zonal accuracies of the EGA plot.

Zone	A	B	C	D	E
Sample percentage	91.8%	5.05%	0%	3.15%	0%

**Table 7:** Evaluation metric values.

Metric	MSE	MAE	RMSE	R^2^	Pearson’s r
Values	110.79	8.51	10.53	0.83	0.91

**Table 8:** Zonal accuracy of the EGA plot.

Zone	A	B	C	D	E
Sample Percentage	80.0%	20.0%	0%	0%	0%

**Table 9:** Evaluation metric values.

Metric	MSE	MAE	RMSE	R^2^	Pearson’s r
Values	258.13	11.6	16.1	0.68	0.85

**Table 10:** Performance comparison with the models in [9].

Model	Pearson’s r	MAE	RMSE	Number of Features	Number of Estimators	Run Time (ms)
XGBoost [9]	**0.89**	11.8	**12.6**	17	**100**	16.087
Random Forest [9]	0.81	13.67	17.4	17	1000	16.475
Ours	0.85	**11.6**	16.1	**1**	**100**	**0.004**

Best values are in boldface font.

## Data Availability

We have created our own dataset for this study. Since further research are on processing, we cannot publish the dataset right now.
